# A rapid and robust method for simultaneously measuring changes in the phytohormones ABA, JA and SA in plants following biotic and abiotic stress

**DOI:** 10.1186/1746-4811-4-16

**Published:** 2008-06-30

**Authors:** Silvia Forcat, Mark H Bennett, John W Mansfield, Murray R Grant

**Affiliations:** 1 Biology Division, Imperial College London, Exhibition Road, London SW7 2AZ, UK; 2 School of Biosciences, University of Exeter, Stocker Road, Exeter, EX4 4QD, UK

## Abstract

We describe an efficient method for the rapid quantitative determination of the abundance of three acidic plant hormones from a single crude extract directly by LC/MS/MS. The method exploits the sensitivity of MS and uses multiple reaction monitoring and isotopically labelled samples to quantify the phytohormones abscisic acid, jasmonic acid and salicylic acid in Arabidopsis leaf tissue.

## Background

Phytohormones play an important role in mediating host responses to various biotic and abiotic stresses such as pathogen challenge, insect herbivory, drought, cold and heat stress. Traditionally, salicylic acid (SA) and jasmonic acid (JA) have been, respectively, associated with resistance to biotrophic and necrotrophic pathogens (reviewed in [1,2]. Although classical SA and JA responsive molecular markers indicate that these phytohormones function antagonistically, recent studies suggest that both the timing and amplitude of hormonal signals play key roles in determining the final pathological phenotype [3,4].

Emerging evidence suggests that a key strategy of plant pathogens is to modify plant hormone levels to promote pathogenicity. Consequently, pathogens have evolved complex repertoires of effector proteins whose functions include modulation of basal phytohormone levels during development of disease. For example, during foliar infection, the hemibiotrophic bacterial pathogen, *Pseudomonas syringae *pv. *tomato *DC3000, delivers ~30 effector proteins into the plant cell [5]. Experimental data suggest they act with a surprising degree of redundancy to modify host signalling pathways, and one clear strategy is to suppress or modify plant hormone responses [6,7].

Recently, the stress hormone, abscisic acid (ABA), better known for its role in response to drought stress and maintenance of seed dormancy (reviewed by (8) has been demonstrated to influence plant pathogen interactions [9-12]. Emerging evidence suggests there are most likely antagonistic interactions between ABA and, JA/ET (ethylene) [13] or SA, signalling pathways depending upon the lifestyle of the infecting pathogen. Thus it is important to be able to measure changes in endogenous concentrations of these hormones at different stages of the infection process. Moreover there is an increasing interest in crosstalk between biotic and abiotic stress pathways [14], how plants prioritize their responses under a given stress and how plants respond to multiple stresses. Plants clearly use phytohormonal signals in a combinatorial manner to achieve distinct outcomes yet actual levels of individual hormones are seldom measured, and if so, only a single hormone is usually quantitated. However the evidence for perturbation of one hormone pathway can having profound effects on synthesis and accumulation of other hormones is considerable [15]. Conventional methods for measuring the hormones such as using enzyme-linked immuno-sorbent assay (ELISA), high-performance liquid chromatography (HPLC) or gas chromatography/mass spectrometry (MS) methods are of limited sensitivity or require a lengthy derivatisation process. Recently we have used C18 solid phase extraction columns for reliable measurements of the acidic hormones SA, ABA and JA [10,16], however the methodology is time-consuming. Currently, no single method appears to be suitable for the range of hormones implicated in plant pathogen interactions.

We therefore sought to develop a robust quantitative analysis using crude soluble plant extracts by exploiting the high sensitivity of LC/MS. Here we present a method for determining ABA, JA and SA from a single extract that is rapid, accurate, technically simple and requires minimal amounts of tissue. The nature of the method lends itself to high throughput phytohormone determination from time-delimited sampling of plant responses in which these hormones are suspected to participate. This method provides several advantages over previously published methods which individually measure ABA, JA and SA [17-19] as these approaches require time-consuming additional steps such as partitioning of the extracts, solvent evaporation by the use of a rotary evaporator, drying of the sample under N_2 _and resuspension of the residue. Such manipulations compromise the speed of the process, increase potential technical error and restrict its use as a high throughput method. Moreover, this is also the first report where these three acidic hormones are accurately measured from a single extract.

## Methods

While, plant hormones such as ABA has been measured individually in crude extracts, [18] no one method has been published that allows simultaneous simple, rapid and accurate measurement of the three acidic hormones, JA, SA and ABA via LC/MS. We therefore developed a method with an extraction solvent that allowed the reproducible and stable extraction of the analytes of interest from relatively small amounts of starting material as well as the ability to inject directly relatively large volumes of the sample whilst retaining good peak shapes. While here we report characterization of this method on Arabidopsis thaliana leaves, this method is equally applicable to other plant species such as tomato (M. Grant unpublished).

Plants were grown for four to five weeks in a controlled environment chamber under short days (10 h), 70% humidity as previously described [20].

Pathogen or abiotic stressed plant material was harvested into liquid nitrogen and freeze dried. Samples were next placed in a 2 ml microfuge tube and ground in a bead beater (Qiagen or equivalent) with 3 mm tungsten beads at 25 Hz/s for 3 min. Ten milligram of powdered tissue (~110 mg fresh weight, or equivalent to approximately two fully expanded Arabidopsis leaves) was weighed into a new 2 ml microfuge tube and extracted with 400 μl of 10% methanol containing 1% acetic acid to which internal standards had been added (1 ng of ^2^H_6 _ABA, 10 ng of ^2^H_2 _JA and 13.8 ng ^2^H_4 _SA). Each treatment also included an extraction control containing no plant material. A 3 mm tungsten bead was placed in each microfuge tube and samples were extracted in the bead beater for 2 min at 25 Hz/s, placed on ice for 30 min then centrifuged at 13,000 g for 10 min at 4°C. The supernatant was carefully removed and the pellet re-extracted with 400 μl of 10% methanol containing 1% acetic acid. Following a further 30 min incubation on ice the extract was centrifuged and the supernatants pooled. The two extractions resulted in 90–95% recovery of the targeted analytes.

Samples (50 μl) were then analysed by HPLC-electrospray ionisation/MS-MS using an Agilent 1100 HPLC coupled to an Applied Biosystems Q-TRAP 2000 (Applied Biosystems, California, USA). Chromatographic separation was carried out on a Phenomenex Luna 3 μm C18(2) 100 mm × 2.0 mm column, at 35°C. The solvent gradient used was 100%A (94.9% H_2_O: 5% CH_3_CN: 0.1% CHOOH) to 100%B (5% H_2_O: 94.9% CH_3_CN: 0.1% CHOOH) over 20 min. Solvent B was held at 100% for 5 min then the solvent returned to 100% A for 10 min equilibration prior to the next injection. The solvent flow rate was 200 μl/min. To reduce contamination of the MS, the first 2 min of the run was directed to waste using the inbuilt Valco valve.

Analysis of the compounds was based on appropriate Multiple Reaction Monitoring (MRM) of ion pairs for labelled and endogenous JA, SA and ABA using the following mass transitions; ^2^H_2_-JA 211 > 61, JA 209 > 59, ^2^H_4 _SA 141 > 97, SA 137 > 93, ^2^H_6 _ABA 269 > 159, ABA 263 > 153, SA-glyc 299 > 93.

The MS was operated in the negative mode using Turbo-Ionspray™ as the ion source. Optimal conditions were determined using the Quantitative Optimisation feature of the Analyst software both by infusing standards into the MS by syringe pump and injecting standards into a 200 μl/min flow of 50% Solvent A/50% Solvent B.

The optimised conditions were as follows: Temperature 400°C, Ion source gas 1 50 psi, Ion source gas 2 60 psi, Ion spray voltage -4500 V, curtain gas 40 psi, CAD gas setting 2; the DP (-25 V), EP (-9) and CEP (-2) were held constant for all transitions. Collision energies (CE) and dwell times (DT) were specific for each compound/internal standard pair, the parameters used were JA CE-25, DT 100 ms, ABA CE-17, DT 250 ms and SA CE-38, DT 50 ms. Data were acquired and analysed using Analyst 1.4.2 software (Applied Biosystems).

Hormones were determined in three independent samples for each treatment or timepoint.

## Results and Discussion

### Reproducibility of the phytohormone extraction method

**Figure 1:**
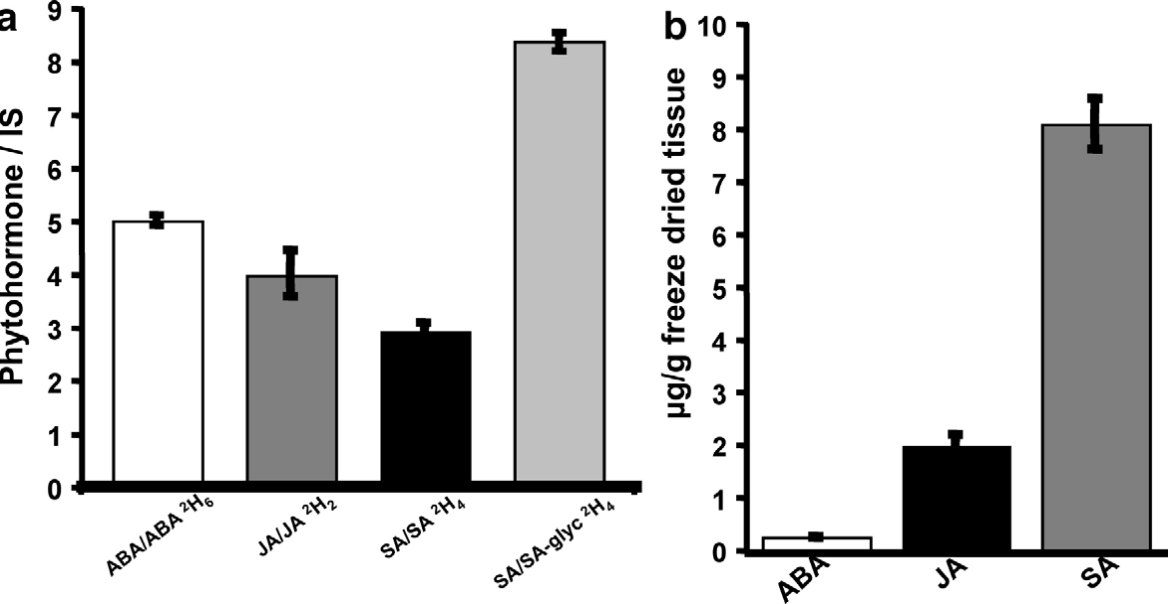
**The phytohormone extraction method is reproducible**. (a) The abundance of the phytohormones ABA, JA, SA and the SA glycoside expressed as a ratio of the added internal standard. Standard error for the seven replicates is less than 10% of the mean value. Glycosylated derivatives can also be determined by ratioing relative to the unglycosylated internal standard. (b) Determination of the absolute value of phytohormones present in the stressed tissue demonstrates that this method can accurately capture the dynamic range of phytohormones with small amounts of starting material (10 mg).

To provide material with representative amounts of the three phytohormones, six leaves on four, 5-week old plants were first wounded with a plastic pipette tip (induction of JA biosynthesis) and then left to dessicate for 2 h (induction of ABA). Material was harvested in aluminium foil, frozen immediately in liquid nitrogen, crushed to generate a homogeneous mixture and freeze dried. Seven 10 mg samples of freeze dried material were extracted after the incorporation of deuterated hormone standards and each hormone was expressed as a ratio of phytohormone to deuterated internal standard (IS). This method produces highly reproducible quantitation as determined by the mean ratios of phytohormone/IS and associated standard errors (maximum of 10% of replicate means) as shown in Table 1. The absolute amounts of each hormone as determined in the freeze dried tissue are presented as ratios of phytohormone/IS (Fig. 1a) or absolute amounts of phytohormone/g freeze dried tissue (Fig. 1b), demonstrating that this method successfully captures the dynamic range of stress related hormone changes from small tissue samples. The method also allows determination of glycosylated derivatives of salicylic acid by quantitation relative to the ^2^H_4 _SA standard (Fig. 1a).

**Table 1:** Hormone extraction reproducibility in technical replicate of extracts of wounded and desiccated tissue.

**Phytohormone/IS**	**Mean**	**Standard error**
ABA/^2^H_6_ABA	5.02	± 0.09
JA/^2^H_2_JA	3.99	± 0.40
SA/^2^H_4_SA	2.94	± 0.17
SA-glyc/^2^H_4_SA	8.39	± 0.17

### Reproducibility of LC/MS measurements

To test the reproducibility of the hormone measurements with this LC/MS method the wounded and desiccated material described above was repeatedly injected (10 times). Table 2 shows all hormone measurements were highly reproducible, with a Relative Standard Deviation (% RSD) for quantification under 4% and for Retention Time under 0.18%.

**Table 2:** LC-MS reproducibility in hormone determination following 10 replicate injections of a stress-treated extract

**Analyte/IS**	**RSD % Quantification**	**RSD % Retention Time**
ABA/^2^H_6_ABA	3.62	1.60 e-14
JA/^2^H_2_JA	1.76	1.40 e-14
SA/^2^H_4_SA	3.80	1.60 e-14
SA-glyc/^2^H_4_SA	2.49	0.18

### Sample stability

It is important to develop a method that is not only facile and robust, but also amenable to high throughput screening, for example screening of mutant and knockout lines or chemical banks for altered phytohormone profiles. To test the stability of the extracts three samples of wounded and dessicated tissue were injected and analysed. These samples remained in the autosampler (6°C) for 48 h and were then reinjected. Table 3 summarizes the ratio of phytohormone to internal standard at t = 0 and t = 48 h. No significant degradation of the sample was detected over the 48 h period, indicating that this method can be used to prepare and screen at least one hundred samples in one run.

**Table 3:** Phytohormones remain stable two days after extraction.

**Analyte/IS**	**RSD % Quantification**	**RSD % Retention Time**
ABA/^2^H_6_ABA	4.05	1.70 e-14
JA/^2^H_2_JA	22.70	0.00
SA/^2^H_4_SA	17.00	1.70 e-14
SA-glyc/^2^H_4_SA	3.92	0.09

**Figure 2:**
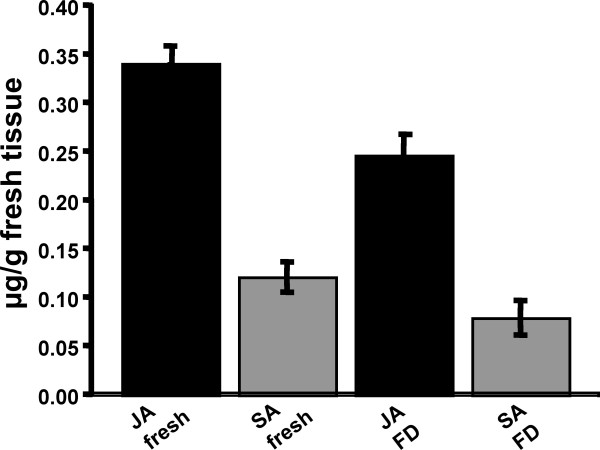
**Comparison of absolute yields from freeze dried (FD) or fresh frozen Arabidopsis leaf material**. Yields of both JA and SA were consistently higher using fresh frozen material, probably due to a combination of both analyte insolubilisation or volatilisation during the freeze drying process.

To determine whether freeze drying adversely affected phytohormone content, fresh frozen and freeze dried material were compared. Ten replicate samples, each containing three expanded leaves from a 5 week old plant were prepared. Each replicate was weighed and one set of five replicates was freeze dried and ground in the bead beater while the other five samples were frozen in liquid nitrogen and then ground to a fine powder in liquid nitrogen. All extraction volumes were identical for each set of replicates. Absolute levels of JA and SA were measured and expressed as gFW of tissue, based upon original fresh weight measurements (Fig. 2). Comparison of the levels of phytohormones from fresh and lyophilized material, showed that yields were consistently lower, about 25%, less from the freeze dried tissue. This result illustrates that freeze drying process reduces phytohormone extraction yields using this method. However, in many instances the convenience of freeze dried material, especially when weighing large replicated samples sets, more than compensates for these reduced yields. This compares favourably with a 50% decrease in the absolute amounts of SA but no change in JA levels reported when freeze dried extractions from cucumber were compared to those from the equivalent amount of fresh tissue [19].

### Linearity of response

**Figure 3:**
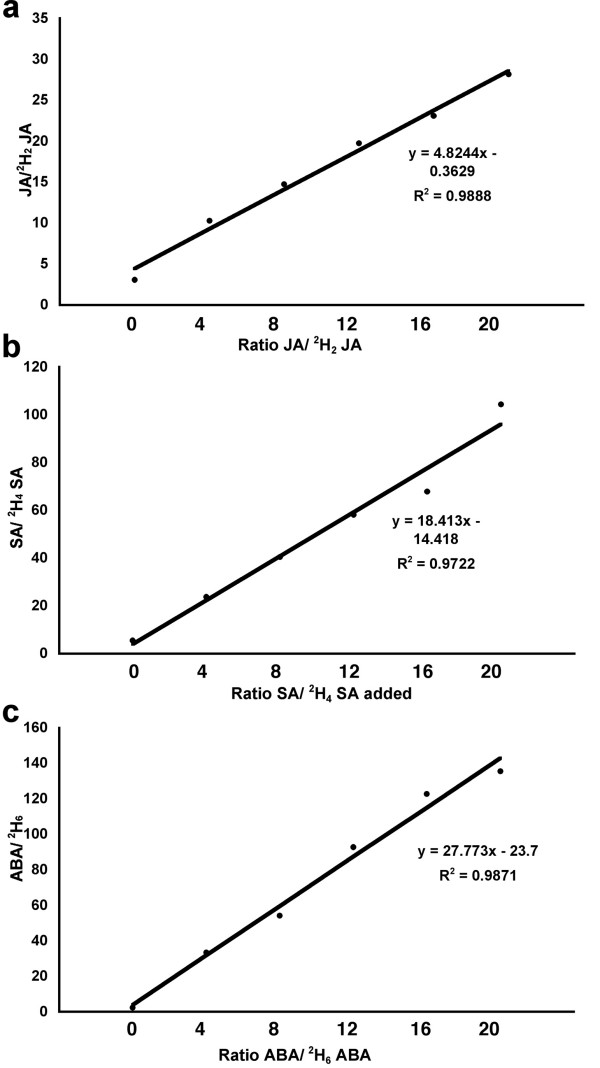
**Linearity of detection of phytohormones**. To ensure the method is capable of capturing the range of differences in phytohormones expected during stress associated experiments, five diluted deuterated standards were compared to unlabelled "stressed controls" in ratios indicated. a-c demonstrates that for ABA, JA and SA respectively, there is a statistically significant linear increase in abundance of the expected ion over a 20 fold range.

Because different plant extracts will have differing baseline hormone levels it is important to demonstrate that this method can accurately measure a proportional increase in the discriminatory m/z ion signal with increasing amounts of each hormone. To address this question a bulk sample derived from combining eighteen 10 mg extracts was prepared, and then realiquoted into eighteen 500 μl samples of which triplicate aliquots were subject to the following six treatments. Treatment 1 comprised control untreated samples. Treatments 2–6 had, respectively, the following ratios of deuterated standards compared to unlabelled phytohormone added;, 1:4, 1:8, 1:12, 1:16 and 1:20. Plotting the ratio of non-deuterated to deuterated standards for JA, SA and ABA (Fig. 3) shows that there was a linear increase in the m/z "area under the curve" signal across the range of concentrations that we routinely expect to be detected in various plant-pathogen interactions and abiotic stresses. The limits of detection based on a ratio of 1:3 signal to noise (LOD) calculated from the standard addition curves for JA, ABA and SA, were 0.22, 0.05 and 0.9 ug/g of freeze dried tissue respectively. The limits of quantification 0.45, 0.11 and 1.9 for JA, ABA and SA respectively, were calculated in the same way as the LOD, based on a ratio 1:6 signal to noise.

**Figure 4:**
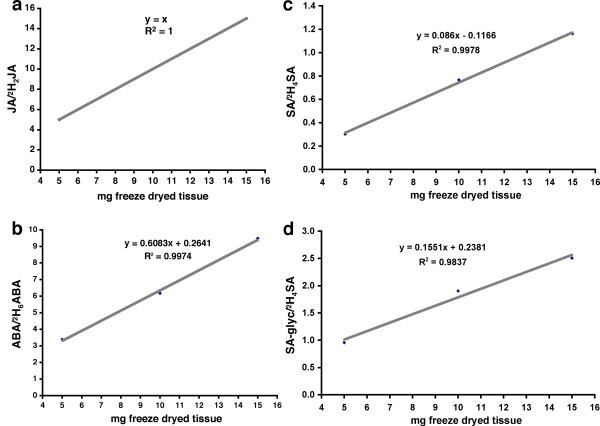
**Phytohormone response metrics**. To determine whether 10 mg was sufficient sample to elicit a linear response in LC/MS signal, phytohormones were determined in 5, 10 and 15 mg amounts of freeze dried material. ABA, JA and SA and SA-glyc (a-d respectively) all show a linear response with increasing amounts of sample (R^2 ^> 0.997).

We next confirmed that the amount of sample used in this method was within the linear range whereby an increase in sample amount is proportional to an increase in LC/MS signal for each of the analysed hormones. Replicate extractions of 5, 10 and 15 mg aliquots of freeze dried leaves were analysed by LC/MS and phytohormone/IS ratios determined. Fig. 4 clearly shows that all three phytohormones produced a linear response with increasing amounts of sample (R^2 ^> 0.997).

**Figure 5:**
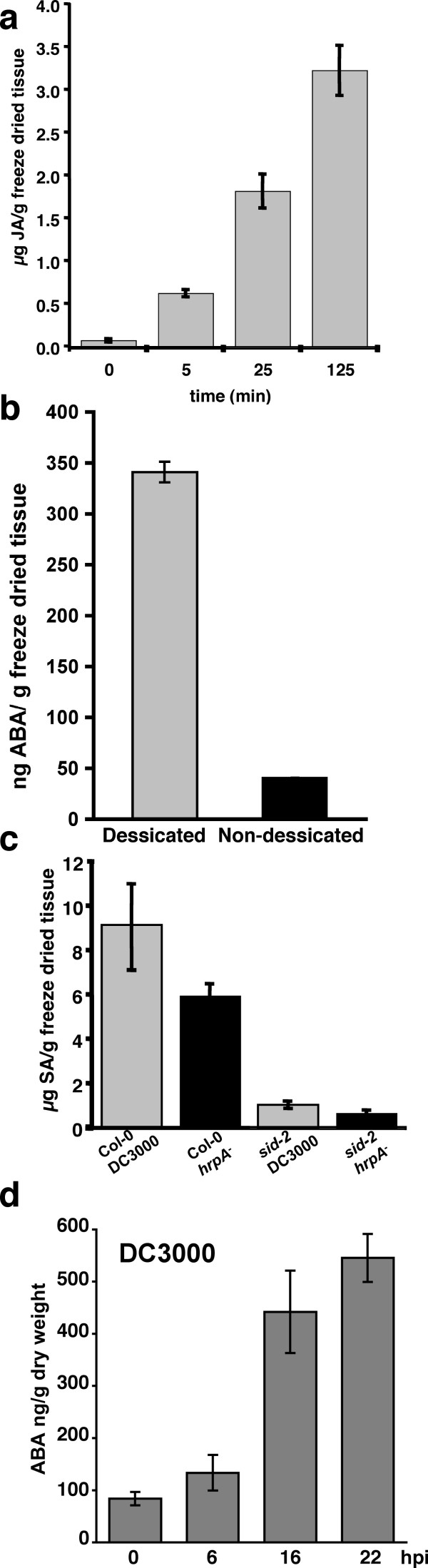
**The extraction method is capable of capturing the dynamic response of phytohormones to inducing stresses**. JA, ABA and SA levels were determined following stresses designed to elevate specific levels of each hormone (a-c). (a) Following wounding by mechanical damage JA levels increase 8 fold within 5 minutes and increase over the following 2 h. (b) Two hours desiccation of detached leaves (at 60% RH) is sufficient to increase foliar ABA levels 8 fold. (c) Challenge with the virulent bacterial pathogen, DC3000 or the DC3000 *hrp *mutant elicits increases in SA levels 21 hpi in wild-type but not the SA biosynthetic mutant, *sid2*. (d) Challenge with virulent DC3000 induces ABA in Arabidopsis leaves within 6 hours post inoculation (hpi).

To demonstrate that this method could capture dynamic changes in phytohormones we applied treatments designed specifically to modulate levels of each hormone. We first analysed changes in JA levels following leaf wounding. Fig. 5a shows an ~8 fold increase in JA within 5 min of wounding an Arabidopsis leaf with a micropipette tip. Wound induced JA levels remained significantly elevated for more than 2 h following treatment.

ABA levels were induced by leaving a detached leaf to desiccate at room temperature (~22°C, 60% relative humidity) for 2 h. ABA levels were determined relative to adjacent attached leaves (Fig. 5b). ABA levels are generally undetectable in leaves of Arabidopsis plants grown under controlled environmental conditions unless specially adapted methods are used. By contrast, 2 h of desiccation caused an ~800% increase in ABA levels. The LOD for ABA was ~4 fold that obtained by Lopez-Carbonella & Jaureugi (2005). Their protocol used two different organic extractions and an optimised HPLC method to target ABA. Given the simplicity of our extraction protocol and added ability to detect JA and SA this LOD compares favourably.

Changes in endogenous SA levels were demonstrated by comparing pathogen challenged control plants with the *isochorismate synthase 1 *deficient plant (*sid2*). Col-0 and *sid2 *plants were inoculated with either virulent *P. syringae *pv. *tomato *DC3000 (DC3000) or the type three secretion deficient DC3000 *hrpA *mutant (21). Salicylic acid was determined 21 h post inoculation. As expected, in wild type plants both DC3000 and the *hrp *mutant accumulate significant amounts of SA and SA-glycoside, whereas levels of these metabolites were strongly attenuated in the *sid2 *background (Fig. 5c). By contrast, both ABA (Fig 5d) and JA (data not shown) levels increased following challenge with DC3000 as previously determined using 70% methanol extracts and C18 solid phase extraction columns prior to LC/MS [10].

## Conclusion

We have developed a rapid, high throughput, cost effective method for quantification of the three major stress hormones in *Arabidopsis*. The method requires minimal tissue, is highly reproducible and can accurately measure phytohormones across the expected physiological dynamic range. Moreover, it compares well with other methods that have more complex extraction methods that specifically target the individual hormones, ABA, JA or SA targeted here. The use of freeze dried material promotes ease of handling and automation. Yield decreases associated with freeze drying compared to fresh-frozen material, probably due either to analyte insolubilisation or to volatilization during the freeze drying process, were minimized. This method is equally applicable to fresh or freeze dried tissues and the experimental circumstances will dictate the starting material. In our experience, use of freeze dried tissue is more convenient for scaling up extraction, especially when weighing multiple samples, e.g. during for time course analyses. The advantage of using Multiple Reaction Monitoring is that it is relatively easy to customise runs to identify other discriminatory metabolites, such as aromatic derived secondary compounds, which are readily associated with plant stress responses.

## Authors' contributions

All authors designed the experiments. SF carried out the plant stress experiments and sample processing, MB designed and carried out the mass spectrometry methods. All authors participated in data extraction and statistical analysis. SF and MG wrote the manuscript. All authors read and approved the final manuscript
